# The association of quantitative PSMA PET parameters with pathologic ISUP grade: an international multicenter analysis

**DOI:** 10.1007/s00259-024-06847-y

**Published:** 2024-08-01

**Authors:** Timo F. W. Soeterik, Joris G. Heetman, Rick Hermsen, Lieke Wever, Jules Lavalaye, Maarten Vinken, Clinton D. Bahler, Courtney Yong, Mark Tann, Claudia Kesch, Robert Seifert, Tugce Telli, Peter Ka-Fung Chiu, Kwan Kit Wu, Fabio Zattoni, Laura Evangelista, Emma Segalla, Antonio Barone, Francesco Ceci, Pawel Rajwa, Giancarlo Marra, Elio Mazzone, Jean-Paul A. Van Basten, Harm H. E. Van Melick, Roderick C. N. Van den Bergh, Giorgio Gandaglia, on behalf of the EAU-YAU Prostate Cancer Working Party

**Affiliations:** 1 Department of Urology, St. Antonius Hospital, Koekoekslaan 1, 3435 CM Nieuwegein, The Netherlands; 2 Department of Radiation Oncology, University Medical Center Utrecht, Utrecht, The Netherlands; 3 Department of Nuclear Medicine, Canisius Wilhelmina Hospital, Nijmegen, The Netherlands; 4 Department of Nuclear Medicine, St. Antonius Hospital, Nieuwegein/Utrecht, The Netherlands; 5 Department of Urology, Indiana University Medical Center, Indianapolis, IN USA; 6 Department of Radiology and Imaging Sciences, Indiana University Medical Center, Indianapolis, IN USA; 7 Department of Urology, University Hospital Essen, Essen German Cancer Consortium (DKTK) University Hospital Essen, Essen, Germany; 8 Department of Nuclear Medicine, Inselspital, Bern University Hospital, Bern, Switzerland; 9 Department of Nuclear Medicine, University Hospital Essen, Essen, Germany; 10 S. H. Ho Urology Centre, Department of Surgery, The Chinese University of Hong Kong, Hong Kong, China; 11 Department of Nuclear Medicine and PET, Hong Kong Sanatorium and Hospital, Hong Kong, China; 12 Department of Surgery, Oncology, and Gastroenterology, Urological Unit, University of Padova, Padua, Italy; 13 Department of Biomedical Sciences, Humanitas University, Pieve Emanuele, Milan, Italy; 14 Division of Nuclear Medicine, IRCCS Humanitas Research Hospital, Milan, Italy; 15 Division of Nuclear Medicine and Theranostics, IEO European Institute of Oncology, IRCCS, Milan, Italy; 16 Department of Urology, Comprehensive Cancer Center, Medical University of Vienna, Vienna, Austria; 17 Department of Urology, Medical University of Silesia, Zabrze, Poland; 18 University Hospital S Giovanni Battista, Azienda Ospedaliero Universitaria Città Della Salute E Della Scienza Di Torino, Turin, Italy; 19 Division of Oncology/Unit of Urology, Soldera Prostate Cancer Lab, URI, IRCCS San Raffaele Scientific Institute, Vita-Salute San Raffaele University, Milan, Italy; 20 Department of Urology, Canisius Wilhelmina Hospital, Nijmegen, The Netherlands; 21 Department of Urology, Erasmus Medical Center, Rotterdam, The Netherlands

**Keywords:** PSMA PET/CT, Prostate cancer, Histology, ISUP grade group

## Abstract

**Purpose:**

To assess if PSMA PET quantitative parameters are associated with pathologic ISUP grade group (GG) and upgrading/downgrading.

**Methods:**

PCa patients undergoing radical prostatectomy with or without pelvic lymph node dissection staged with preoperative PSMA PET at seven referral centres worldwide were evaluated. PSMA PET parameters which included SUV_max_, PSMA_volume_, and total PSMA accumulation (PSMA_total_) were collected. Multivariable logistic regression evaluated the association between PSMA PET quantified parameters and surgical ISUP GG. Decision-tree analysis was performed to identify discriminative thresholds for all three parameters related to the five ISUP GGs The ROC-derived AUC was used to determine whether the inclusion of PSMA quantified parameters improved the ability of multivariable models to predict ISUP GG ≥ 4.

**Results:**

A total of 605 patients were included. Overall, 2%, 37%, 37%, 10% and 13% patients had pathologic ISUP GG1, 2, 3, 4, and 5, respectively. At multivariable analyses, all three parameters SUV_max_, PSMA_volume_ and PSMA_total_ were associated with GG ≥ 4 at surgical pathology after accounting for PSA and clinical T stage based on DRE, hospital and radioligand (all *p* < 0.05). Addition of all three parameters significantly improved the discrimination of clinical models in predicting GG ≥ 4 from 68% (95%CI 63 – 74) to 74% (95%CI 69 – 79) for SUV_max_, 72% (95%CI 67 – 76) for PSMA_volume_, 74% (70 – 79) for PSMA_total_ and 75% (95%CI 71 – 80) when all parameters were included (all *p* < 0.05). Decision-tree analysis resulted in thresholds that discriminate between GG (SUV_max_ 0–6.5, 6.5–15, 15–28, > 28, PSMA_vol_ 0–2, 2–9, 9–20 and > 20 and PSMA_total_ 0–12, 12–98 and > 98). PSMA_volume_ was significantly associated with GG upgrading (OR 1.03 95%CI 1.01 – 1.05). In patients with biopsy GG1-3, PSMA_volume_ ≥ 2 was significantly associated with higher odds for upgrading to ISUP GG ≥ 4, compared to PSMA_volume_ < 2 (OR 6.36, 95%CI 1.47 – 27.6).

**Conclusion:**

Quantitative PSMA PET parameters are associated with surgical ISUP GG and upgrading. We propose clinically relevant thresholds of these parameters which can improve in PCa risk stratification in daily clinical practice.

**Supplementary Information:**

The online version contains supplementary material available at 10.1007/s00259-024-06847-y.

## Introduction

The use of PSMA PET/CT to assist primary staging of prostate cancer (PCa) is characterized by a higher sensitivity for the detection of nodal and distant metastasis compared to conventional imaging [1–5]. More recently, PSMA PET-derived quantified parameters have been proposed to improve risk stratification [6]. One of the most extensively investigated quantitative parameter for analysis of tracer uptake includes the standardized uptake value (SUV). SUV_max_ is defined as the SUV of the single voxel in a region of interest that presents the highest uptake on the attenuation-corrected PET image [7]. SUV_max_ has been previously shown to have high reproducibility [8]. Since PSMA expression is observed with the greatest extent and intensity in the highest Gleason primary patterns 4 and 5, SUVmax might improve our ability to risk stratify PCa [9, 10]. Prior studies exploring the association between uptake values have shown that Gleason scores were correlated with the intensity of tracer accumulation in the primary tumor, showing that SUV_max_ among patients with Gleason scores ≤ 7 were significantly lower compared with patients with Gleason scores > 7 [11]. Similarly, [^68^ Ga]Ga-PSMA-11 SUV_max_ was significantly higher among patients with Gleason ≥ 4 + 3 compared with Gleason ≤ 3 + 4 [12]. Besides SUV_max_, other quantitative PSMA PET parameters such as intraprostatic PSMA_volume_ and PSMA_total_ have been reported to be significantly associated with surgical outcomes. PSMA_volume_ is the total quantified PSMA positive volume of the prostate tumor, whereas PSMA_total_ represents the total PSMA accumulation (PSMA_volume_ x SUV_mean_) of the tumor. These parameters could add value to SUV_max_, in terms of PCa prognostication, as they also provide information regarding the size and total uptake of the region of interest. For example, both PSMA_volume_ and PSMA_total_ were associated with lymph node involvement (LNI) at pelvic lymph node dissection [13]. However, it is unclear how these parameters relate to histopathological features such as the International Society of Urological Pathology (ISUP) Grade Group (GG) of the primary tumor, and if these parameters provide additional predictive value to SUV_max_ alone.

Although previous studies confirm the association of quantitative PSMA PET parameters with PCa histopathological findings, reliable and reproducible thresholds to further guide clinical decision-making are lacking. As most prior studies on this subject include single-center, relatively small cohorts, there is an urgent need for studies with larger sample sizes. In addition, the majority of studies describe the use of [^68^ Ga]Ga-PSMA-11, and it is unclear how quantitative parameters and their association with histopathology relate among other radioligands. In the face of such a paucity of data, we sought to evaluate the association between PSMA PET quantitative parameters with disease aggressiveness (namely, pathologic ISUP grade group) in a large international multi-center cohort of PCa patients undergoing radical prostatectomy.

## Methods

### Patient population

Men with histopathologically proven PCa undergoing radical prostatectomy with or without pelvic lymph node dissection and preoperatively staged with PSMA PET/CT in the period 2016 to 2023 at seven tertiary referral centers were included. Patients were excluded if they underwent prior other (systemic) therapy for PCa. Histopathological reporting of the surgical specimen was done by local dedicated uropathologists according to the ISUP guidelines [14].

### PSMA PET/CT procedures

All PSMA PET scans were performed at the tertiary referral centers according to the local protocol. A description of the PET protocols used per hospital is presented in Supplementary Table 1. The inclusion of PET scans performed externally for referred patients was allowed. These PET scans were re-read by the local team. PET images were made from mid-thigh to skull base and combined with a low-dose CT scan or a diagnostic CT scan for anatomical correlation. All PSMA PET scans were evaluated by an experienced nuclear medicine physician (> 5 yr experience and/or > 500 studies) at each referral center. The radioligands used included [^68^ Ga]Ga-PSMA-11, [^18^F]PSMA-1007, [^18^F]DCF-PyL, and [^18^F]-JK-PSMA-7, according to specific center preference. Images were acquired according to European Association of Nuclear Medicine/Society of Nuclear Medicine and Molecular Imaging criteria [15].

### PSMA PET/CT parameters

To collect additional PSMA parameters not standardly reported during routine clinical care, all PSMA PET scans were prospectively reassessed and read by the local nuclear medicine physician or research fellow under the direct supervision of the nuclear medicine staff physician. PSMA parameters were assessed by delineating the PSMA-expressing tumors, which represent the volume of interest, manually within the prostate with the threshold set to SUV_max_ ≥ 4. Neighboring anatomical tissues with high PSMA accumulation (e.g. urinary bladder) were excluded. PSMA parameters calculated one whole-gland level included SUV_max_, PSMA positive volume (PSMA_volume_), and total PSMA accumulation (PSMA_volume_ × SUV_mean_ [of the selected volume of interest] = PSMA_total_).

### Statistical analysis

#### Pairwise comparison of the distribution of PSMA PET/CT parameters per ISUP GG

Since data of PSMA PET parameters were not normally distributed, non-parametric tests were employed. Median values of all three PSMA PET parameters were assessed per surgical ISUP GG, and pairwise comparisons of median values per pathologic ISUP GG were performed. The Kruskall-Wallis test was used to compare > 2 independent groups, including post-hoc pairwise comparisons of all separate ISUP GG (1 to 5) using Dunn’s test and applying the Bonferroni correction.

#### Multivariable logistic regression analyses predicting pathologic ISUP GG ≥ 4

Uni- and multivariable logistic regression analysis assessed the association of SUV_max_, PSMA_volume_, and PSMA_total_ with pathologic ISUP GG ≥ 4 after adjusting for potential confounders. To establish whether the potential association varied among radioligands, multivariable logistic regression analysis was done including patients undergoing PSMA PET with use of either [^68^ Ga]Ga-PSMA-11 or [^18^F]PSMA-1007, adjusting for clinical stage based on DRE, preoperative PSA and hospital. The ROC-derived AUC of models predicting ISUP GG ≥ 4 was calculated before (clinical variables only) and after including PSMA PET parameters.

#### Decision-tree analysis for discerning thresholds related to ISUP GG 1 to 5

We then employed decision tree analysis, a machine learning technique, to identify discriminative thresholds for SUV_max_, PSMA_volume_, and PSMA_total_ related to the five ISUP GGs. The aim of this analysis was to explore presence of cut-offs who are directly proportional to ISUP GG histology. The decision tree model was trained on the dataset, iteratively splitting subclasses based on the values of the continuous variables to create a tree structure, using the CHAID (Chi-square Automatic Interaction Detection) method. To reduce overfitting, tenfold cross validation was employed [16, 17].

#### Association between PSMA PET parameters and GG upgrading and downgrading

The association between SUV_max_, PSMA_volume_, and PSMA_total_ and GG upgrading among patients with biopsy ISUP GG < 5, and downgrading among patients with biopsy ISUP GG > 1, were assessed using univariable and multivariable logistic regression analysis. The thresholds resulting from the decision-tree analysis were explored to assess most optimum cut-offs for the prediction of both upgrading and downgrading.

All statistical analyses were done using SPSS (IBM Corp. Version 25.0. Armonk, NY) and R v4.2.1. (R Project for Statistical Computing, www.r-project.org).

## Results

### Patient baseline characteristics

A total of 605 patients were included per analysis. The median age at surgery was 66 years (IQR 62 – 71) and the median preoperative serum PSA level was 9.5 ng/ml (IQR 6.4 – 16.1). Overall, 2%, 43%, and 56% of patients had EAU low-, intermediate- and high-risk PCa. MRI information (PI-RADS score and staging info) was available in 534 (88%) of patients. Among patients with PI-RADS 3 or higher on MRI, target biopsy was performed in 77% of cases. In the vast majority of cases (95%), radioligands [^68^ Ga]Ga-PSMA-11 (62%) and [^18^F]PSMA-1007 (33%) were used. The median SUV_max_, PSMA_volume_, and PSMA_total_ were 9.8 (IQR 6.1 – 16.4), 4.6 (IQR 1.4 – 10.7), and 29.8 (IQR 8.0 – 77.5), respectively (Table 1). Boxplots of all three parameters per ISUP GG are shown in Supplementary Fig. 1. At final surgical pathology, 136 patients (23%) had ISUP grade ≥ 4, while 29% of men had localized disease (pT2), and extraprostatic extension (pT3a) and seminal vesicle invasion (pT3b) were observed in respectively 49% and 22% (Table 1).

**Table 1:** Baseline characteristics of the included 605 patients

	*N* (%)
Age (yr), median (IQR)	66 (62 – 71)
Weight (kg), median (IQR)	86 (77 – 99)
Hospital	
1	146 (24)
2	214 (35)
3	55 (9)
4	108 (18)
5	46 (8)
6	20 (3)
7	16 (3)
PSA (ng/ml), median (IQR)	9.5 (6.4 – 16.1)
< 10	324 (54)
10–20	174 (29)
> 20	107 (18)
Biopsy ISUP Grade Group	
1	30 (5)
2	147 (24)
3	175 (29)
4	159 (26)
5	93 (15)
Missing	1 (0)
Clinical stage based on DRE	
T1	285 (47)
T2	233 (39)
T3	73 (12)
Missing	14 (2)
EAU risk group	
Low	10 (2)
Intermediate	257 (43)
High	338 (56)
MRI stage	
No visible lesion	19 (3)
T2	281 (46)
T3a	177 (29)
T3b	55 (9)
T4	2 (0)
Missing/no MRI	71 (12)
Biopsy strategy	
Systematic	191 (32)
Target biopsy	73 (12)
Systematic and target biopsy	302 (50)
Missing data	39 (6)
Radioligand	
^68^ Ga-PSMA-11	374 (62)
^18^F-PSMA-1007	200 (33)
^18^F-JK-PSMA-7	22 (4)
^18^F-DCFPyL	6 (1)
Missing	3 (1)
SUV_max_ Median (IQR)	9.8 (6.1 – 16.4)
PSMA_total_ Median (IQR)	4.6 (1.4 – 10.7)
PSMA_vol_ Median (IQR)	29.8 (8.0 – 77.5)
Surgical ISUP grade group	
1	14 (2)
2	226 (37)
3	225 (37)
4	58 (10)
5	78 (13)
Missing	4 (1)
Pathological T stage	
T2	174 (29)
T3a	298 (49)
T3b	131 (22)
T4	2 (0)

^*^Percentages may not equal 100 due to rounding

### Pairwise comparison of the distribution of PSMA PET/CT parameters per ISUP GG

The median values of all three PSMA parameters differed significantly per ISUP GG and were directly proportional in value (Table 2). In the pairwise comparative analysis of each GG pair, SUV_max_ showed highest heterogeneity in the pairwise comparison of median values per ISUP GG, showing significant differences for all GG pairs except GG1 vs GG2, GG3 vs GG4 and GG4 vs GG5 (Table 3). Comparing median values per GG, [^18^F]PSMA-1007 vs. [^68^ Ga]Ga-PSMA-11, PSMA_total_ and SUV_max_ median values per GG showed no significant differences in median values comparing both radioligands. For PSMA_volume_ significant differences in median values were observed for GG2 and GG3 comparing both radioligands (Supplementary Table 2).

**Table 2:** Median values of all three PSMA PET parameters across surgical ISUP grade groups

ISUP GG	SUVmax Median (IQR)	*p*	PSMAvolume Median (IQR)	*p*	PSMAtotal Median (IQR)	*p*
1 (*N* = 14)	6.0 (5.3 – 7.6)	< 0.001	2.5 (1.6 – 6.3)	< 0.001	13.6 (7.4 – 30.0)	< 0.001
2 (*N* = 226)	7.5 (5.2 – 12.7)		3.2 (0.6 – 8.0)		21.7 (2.7 – 60.2)	
3 (*N* = 224)	10.5 (6.5 – 16.9)		4.5 (1.8 – 10.7)		30.7 (10.0 – 75.7)	
4 (*N* = 58)	12.2 (7.3 – 23.8)		6.9 (3.3 – 13.2)		47.0 (17.7 – 104.7)	
5 (*N* = 77)	15.3 (8.7 – 29.5)		10.7 (4.0 – 22.8)		71.9 (24.2 – 166.8)

**Table 3:** Pairwise comparisons of the distribution of all three PSMA PET parameters among surgical ISUP grade groups

	SUVmax	PSMAvolume	PSMAtotal
Pairs	*p*	*p*	*p*
GG1 – GG2	1.00	1.00	1.00
GG1 – GG3	*0.042*	1.00	1.00
GG1 – GG4	*0.005*	0.67	0.28
GG1 – GG5	< *0.001*	*0.048*	*0.011*
GG2 – GG3	*0.001*	*0.041*	*0.043*
GG2 – GG4	< *0.001*	*0.003*	*0.004*
GG2 – GG5	< *0.001*	< *0.001*	< *0.001*
GG3 – GG4	0.991	0.762	0.88
GG3 – GG5	*0.006*	< *0.001*	< *0.001*
GG4—GG5	1.00	1.00	0.786

### Uni- and multivariable regression analysis identifying predictors of ISUP GG ≥ 4

**Fig. 1:**
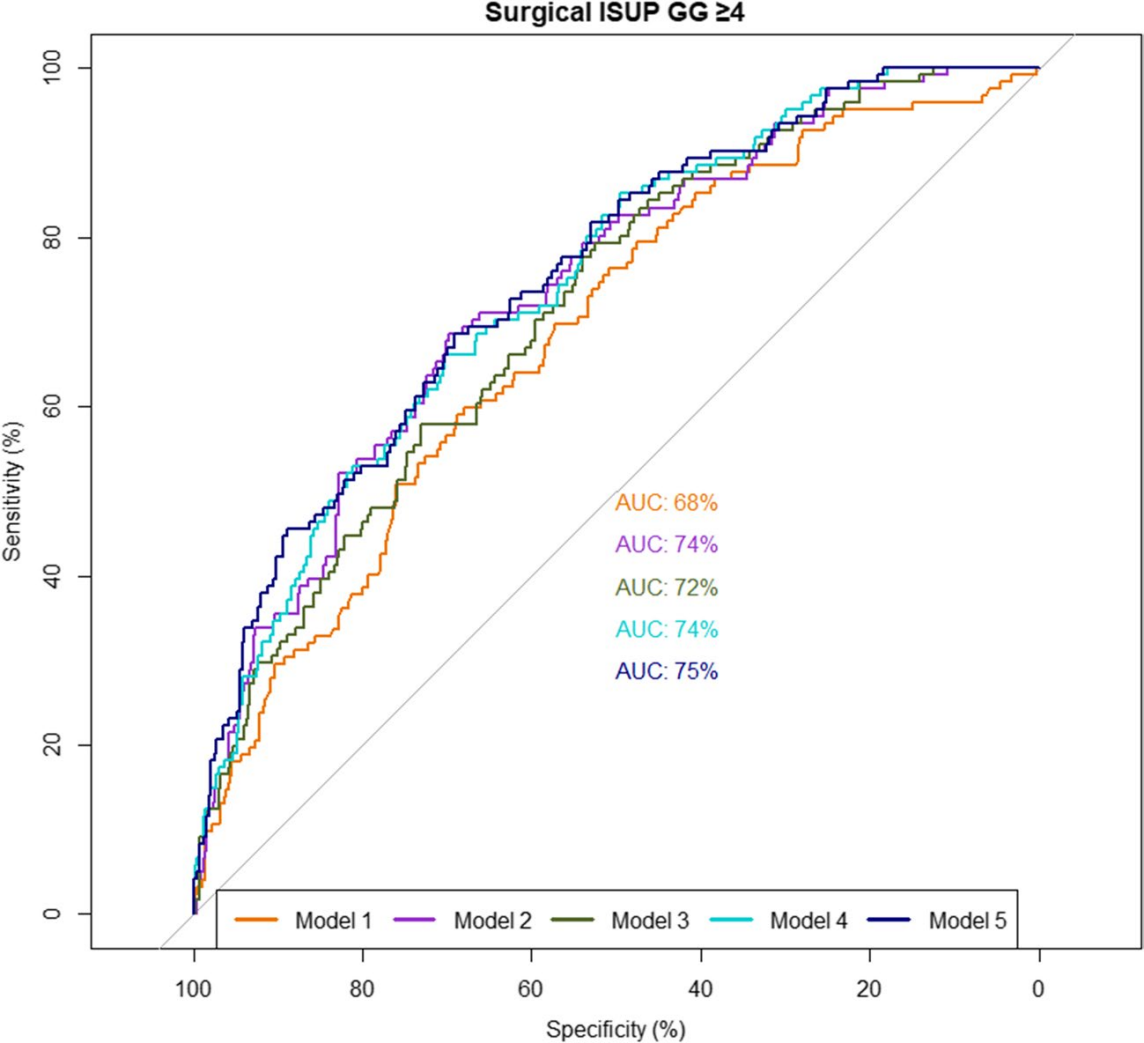
ROC curves of multivariable logistic regression analysis of high-risk surgical ISUP grade group (model 1 to 5) (Table 4)

**Fig. 2:**
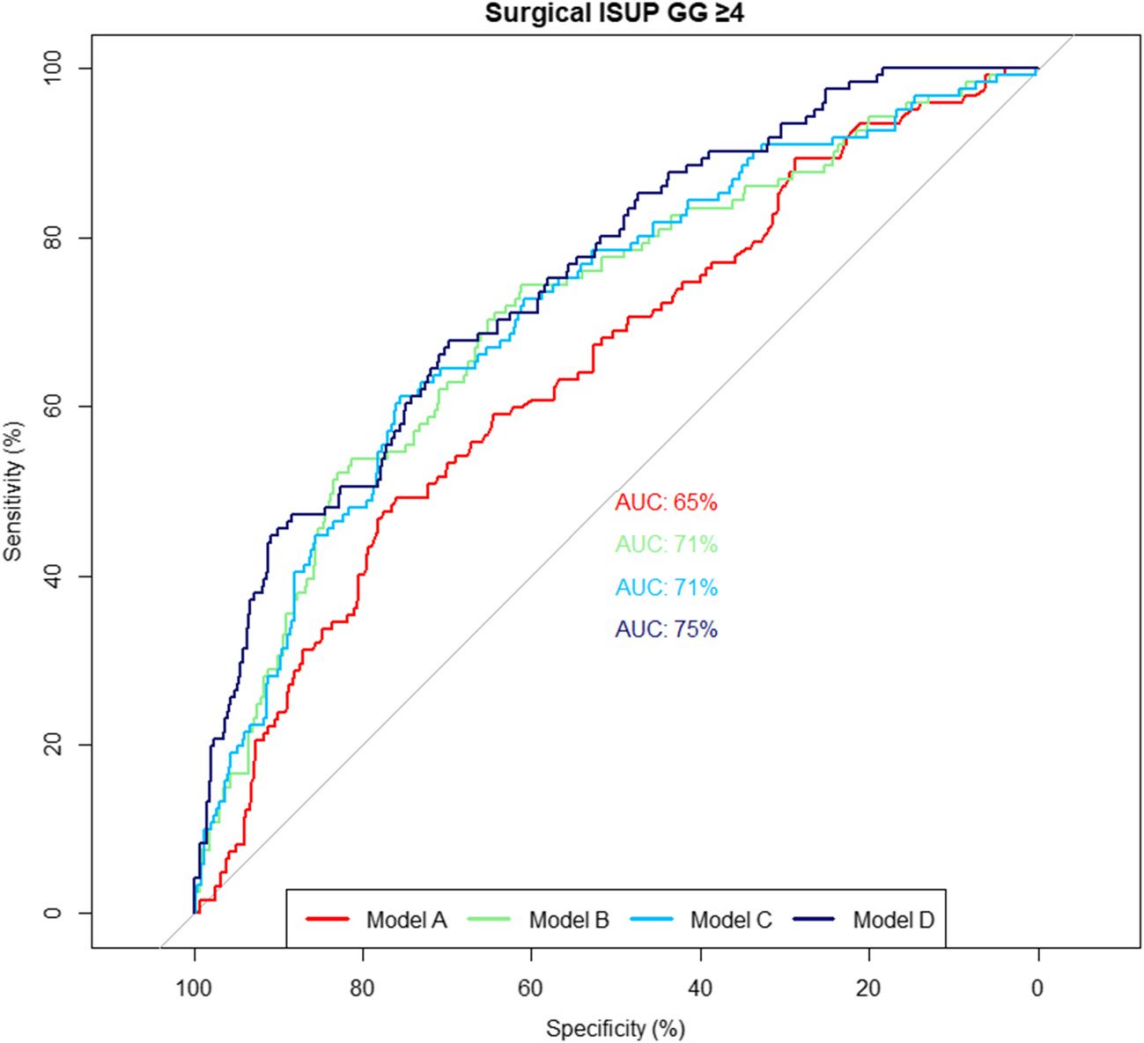
ROC curves of multivariable logistic regression models assessing the impact of hospital and radioligand type on model discrimination (model A to D) (Supplementary Table 4)

At uni- and multivariable logistic regression analyses of all three PSMA parameters separately, SUV_max_, PSMA_volume_ and PSMA_total_ were significantly associated with a pathologic GG ≥ 4 (Supplementary Table 3, all *p* < 0.05). Combining all three parameters in multivariable analysis showed that PSMA_total_ was significantly associated with ISUP GG ≥ 4 (OR 1.005 95%CI 1.002 – 1.007), whereas SUV_max_ and PSMA_volume_ were not (Table 4). PSMA quantified parameters significantly improved the discrimination in terms of AUC of the model with clinical parameters in predicting GG ≥ 4 from 68% (95%CI 63 –74) to respectively 74% (95%CI 69 – 79), *p* < 0.001, for SUV_max_, 72% (95%CI 67 –76), *p* = 0.006, for PSMA_volume_, 74% (95%CI 70 – 79), *p* = 0.003, for PSMA_total_ and 75% (95%CI 71 – 80), *p* = 0.001, with all three parameters included (Table 4 and Fig. 1). Results of additional analyses evaluating the impact of hospital and radioligand on the multivariable models are shown in Supplementary Table 4. When excluding hospital as a covariate (model B), SUV_max_ remained significantly associated with ISUP GG ≥ 4 (OR 1.031, 95%CI 1.009 – 1.054). While addition of hospital as a covariate resulted in a significant increase in AUC from 71% (Model B) to 75% (Model D), *p* = 0.01), accounting for radioligand as a covariate did not significantly change AUC (71% vs 71%, *p* = 0.6) (Supplementary Table 4 and Fig. 2).

**Table 4:** Multivariable logistic regression analysis of high-risk surgical ISUP grade group

	Model 1 Clinical OR (95%CI)	Model 2 SUV_max_ OR (95%CI)	Model 3 PSMA_volume_ OR (95%CI)	Model 4 PSMA_total_ OR (95%CI)	Model 5 All OR (95%CI)
SUV_max_		1.046 (1.025 – 1.066)			1.020 (0.997 – 1.044)
PSMA_volume_			1.022 (1.007 – 1.038)		0.997 (0.980 – 1.014)
PSMA_total_				1.005 (1.003 – 1.008)	1.005 (1.002 – 1.007)
Radioligand					
[^68^ Ga]Ga-PSMA-11		Ref	Ref	Ref	Ref
[^18^F]PSMA-1007		0.56 (0.24 – 1.30)	0.64 (0.28 – 1.49)	0.66 (0.28 – 1.54)	0.64 (0.26 – 1.50)
PSA	1.00 (0.98 – 1.01)	0.99 (0.97 – 1.003)	0.99 (0.97 – 1.01)	0.97 (0.95 – 0.99)	0.97 (0.95 – 0.99)
Clinical stage					
T1	Ref	Ref	Ref	Ref	Ref
T2	1.59 (0.99 – 2.54)	1.61 (0.99 – 2.62)	1.41 (0.87 – 2.28)	1.58 (0.96 – 2.59)	1.63 (0.99 – 2.69)
T3	2.87 (1.55 – 5.32)	2.77 (1.47 – 5.22)	2.65 (1.41 – 4.95)	2.71 (1.42 – 5.15)	2.74 (1.43 – 5.23)
Hospital					
1	Ref	Ref	Ref	Ref	Ref
2	1.92 (1.09 – 3.39)	2.80 (1.06 – 7.38)	2.58 (0.98 – 6.80)	2.35 (0.88 – 6.28)	2.34 (0.87 – 6.37)
3	2.66 (1.28 – 5.52)	2.44 (1.14 – 5.24)	2.68 (1.25 – 5.72)	2.47 (1.14 – 5.33)	2.32 (1.07 – 5.05)
4	0.43 (0.17 – 1.06)	0.29 (0.11 – 0.78)	0.38 (0.15 – 0.95)	0.10 (0.02 – 0.38)	0.09 (0.02 – 0.39)
5–6-7	2.10 (1.05 – 4.20)	1.78 (0.87 – 3.68)	1.53 (0.71 – 3.30)	1.75 (0.85 – 3.61**)**	1.73 (0.80 – 3.74)
**AUC (%) (95%CI)**	**68 (63 – 74)**	**74 (69 – 79)**	**72 (67 – 76)**	**74 (70 – 79)**	**75 (71 – 80)**

### Decision-tree analysis for discerning thresholds for all parameters related to ISUP GG 1 to 5

**Fig. 3:**
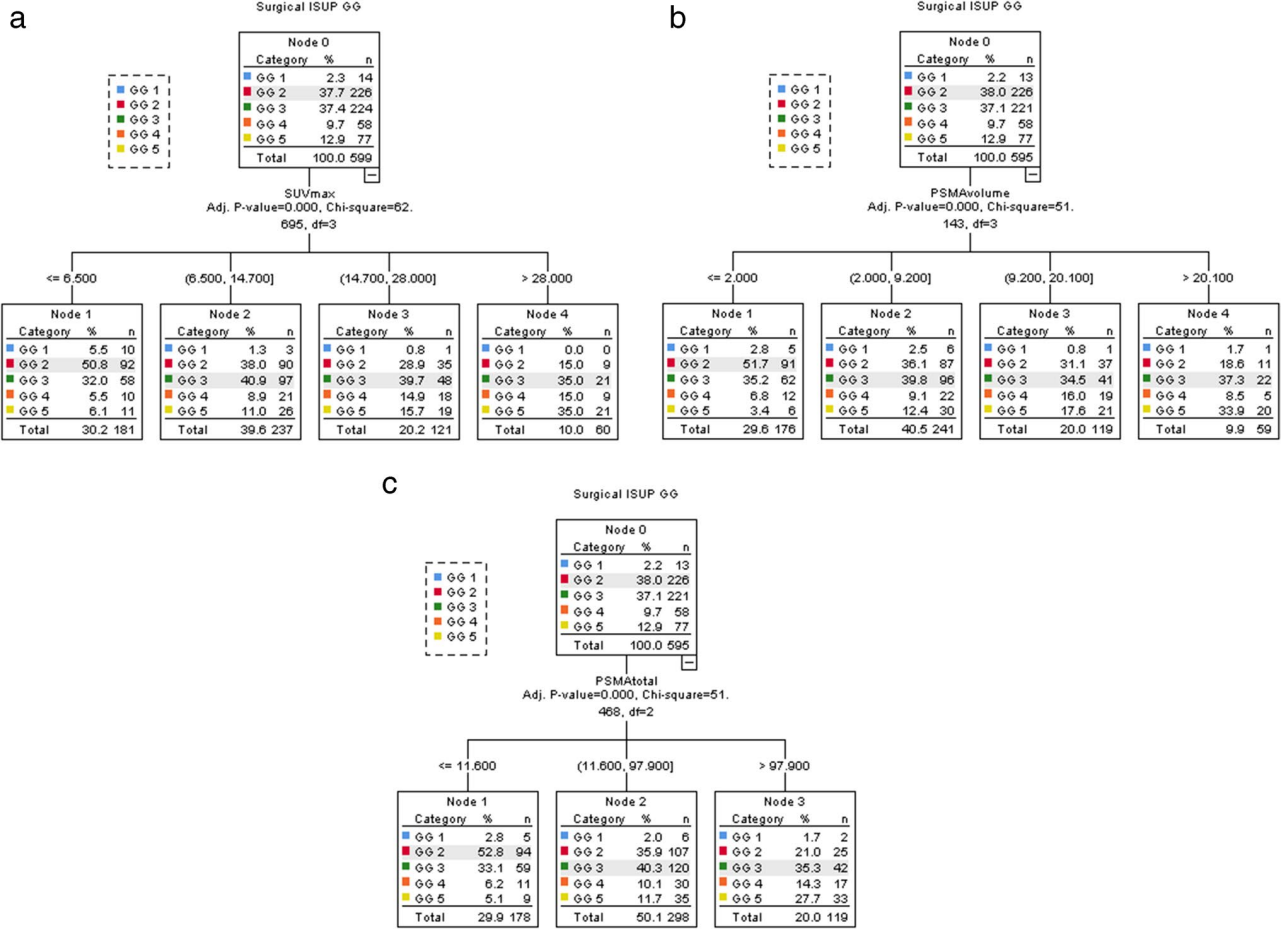
Results of decision tree analysis of SUV_max_ (a), PSMA_volume_ (b), PSMA_total_ (c), and ISUP surgical grade group

Decision-tree analysis resulted in thresholds that discriminate between GG (SUV_max_ 0–6.5, 6.5–15, 15–28, > 28, PSMA_volume_ 0–2, 2–9, 9–20 and > 20 and PSMA_total_ 0–12, 12–98 and > 98). For all three parameters, an absolute increase in proportion of patients with ISUP grade 4 and 5 was observed, directly proportional with PSMA parameters values for each node. An inversely proportional association was observed for proportions of patients with ISUP grade 1 and 2, whereas proportions of patients with ISUP grade 3 remained stable among nodes (Fig. 3a, b, and c.).

### Association between PSMA PET parameters and GG upgrading and downgrading

Upgrading and downgrading were observed in respectively 97 (16%) and 207 (35%) patients. As shown in Table 5, upgrading occurred most frequently in ISUP grade group 1 (*n* = 22, 73%), which included upgrading to GG2 in 53%, GG3 in 17% and GG5 in 5% of cases, respectively. On multivariable analysis including all three PSMA parameters, PSMA_volume_ was significantly associated with GG upgrading (OR 1.027 95%CI 1.007 – 1.049), whereas SUV_max_ and PSMA_total_ were not (Supplementary T﻿able 6). Among patients with biopsy GG1-3 (*n* = 352), upgrading to GG ≥ 4 was observed in 23 (7%) of patients. In patients with biopsy GG1-3, PSMA_volume_ ≥ 2 was significantly associated with higher odds for upgrading to ISUP GG ≥ 4, compared to PSMA_volume_ < 2 (OR 6.36, 95%CI 1.47 – 27.6). PSMA_volume_ was also the only PSMA parameter significantly (inversely) associated with GG downgrading on multivariable analysis (Supplementary Table 8). Among patients with biopsy GG ≥ 4 (*n* = 248), 44 patients (18%) experienced downgrading to GG ≤ 2. Patients with biopsy GG ≥ 4 and PSMA_volume_ ≥ 2, had significantly lower odds (OR 0.42 95%CI 0.21 – 0.87) for downgrading to GG ≤ 2, compared with those with PSMA_volume_ < 2.

**Table 5:**
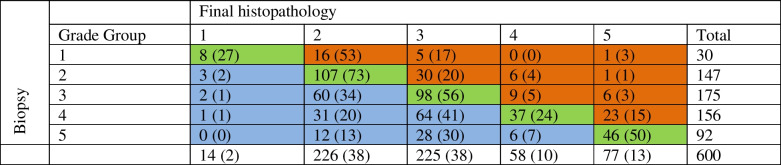
Crosstabulation ISUP grade group at biopsy versus final histopathology after radical prostatectomy

## Discussion

Although previous studies proposed an association between PSMA PET quantitative parameters and disease aggressiveness, their results are poorly generalizable due to the inclusion of small sample sizes and the lack of a comprehensive assessment of all available tracers. As such, we aimed to evaluate the association between PSMA PET quantitative parameters with surgical ISUP GG in a large multi-center cohort of PCa patients undergoing RP treated worldwide. Our multicenter analyses allowed us to propose a clinically relevant subclassification of SUV_max_, PSMA_volume_ and PSMA_total_ associated with ISUP GG ≥ 4 at histopathological evaluation after RP. Analyses of their median values per ISUP GG revealed that these are directly proportionally associated with ISUP grading. SUV_max_ had the best discriminative ability at pairwise ISUP GG comparative analysis. Multivariable analyses including all three PSMA PET parameters, showed that PSMA_total_ was significantly associatied with GG ≥ 4, whereas PSMA_volume_ was associated with upgrading and downgrading_._ Our findings have clinical implications, as our proposed subclassification per parameter can assist PCa risk stratification and guide clinical decision-making.

Previous studies proposed that PSMA parameters can be used to discriminate PCa from benign tissue. For example, Jiao and colleagues evaluated 135 patients who underwent [^68^ Ga]Ga-PSMA-11 PET/CT and showed that using SUV_max_ with a cutoff value of 5.30 can assist with discriminating clinically significant PCa from benign prostatic diseases [18]. Fendler and colleagues proposed an optimal SUVmax cutoff of 6.5 for discrimination between histopathology-positive segments from histopathology-negative segments (AUC 0.84, *p* < 0.001) [19]. In the PRIMARY study, Emmet and colleagues found PSMA intensity to be associated with both PI-RADS and biopsy grade (*p* < 0.001). They also reported a median PSMA SUV_max_ for men without cancer on biopsy of 4.0 (interquartile range [IQR]: 3.4–5.1) versus 12.3 (IQR: 6.3–15.6) for ISUP grade group 5 malignancy [20]. Our analyses suggest that SUV_max_ can assist with discrimination of lower ISUP grades 1, 2 and 3 from the most aggressive ISUP GG 4 and 5. Our findings regarding SUV_max_ are also concordant with those reported by Xue and colleagues, who found that median SUV_max_ was directly proportionally related to percentage of Gleason 4 pattern present in prostate segments after prostatectomy. SUVmax was a fair discriminator of > 50%, > 20% and > 10% Gleason pattern 4 per segment, with AUCs of 78%, 74% and 74%, respectively [21]. In addition, our study showed the added value of PSMA_total_, which remained associated with ISUP GG ≥ 4 when adjusted for SUV_max_ and PSMA_volume_ and other confounders including hospital.

The clinical relevance of relating PSMA PET parameters to ISUP GG at surgical pathology perhaps mostly entails prediction of upgrading. The underlying hypothesis is that in the presence of discrepancy between relatively high uptake values of the tumour at PSMA PET/CT and low ISUP GG on biopsy (e.g. ISUP GG1), there might be an underestimation of histopathological grading. In previous relatively smaller series, SUV_max_ ≥ 5.6 was proposed as the only independent predictor of pathological upgrading from ISUP GG1 to GG ≥ 4, adjusting for maximal tumor core involvement and PI-RADS score of the mpMRI index lesion [22]. Although this analysis should be interpreted cautiously due to the risk of overfitting given the low event-per-variable rate, it suggests the added value of SUVmax for risk stratification. Demirci and colleagues studied 141 patients undergoing RP and proposed that SUV_max_ values significantly correlate to ISUP GG of the primary tumor. In particular, SUV_max_ of high-risk patients were significantly higher than those of low-risk patients. Using a SUV_max_ cut-off of 9.1 would have predicted upgrading from GG1-2 to GG3-4–5 in 63% of the patients [23]. Raveenthiran and colleagues reported that in patients with ISUP GG ≤ 2 and a SUV_max_ < 5, only 10% were upgraded to ISUP GG ≥ 3 at surgical pathology, compared to 90% if the SUV_max_ was > 11 [24]. In this study, although SUV_max_ was a significant discriminator in terms of median values comparing ISUP GG pairs, PSMA_volume_ was significantly associated with both upgrading and downgrading on multivariable analysis. Our subgroup analysis revealed that a threshold of 2 could assist in predicting upgrading to GG ≥ 4 for patients with biopsy GG ≤ 3, as well as the likelihood of downgrading to GG ≤ 2 in patients with GG ≥ 4. However, it should be emphasized that this is a retrospective cohort of patients treated in routine clinical care who had the indication for undergoing a staging PSMA PET/CT, including mostly unfavorable intermediate-risk and high-risk patients (71% had GG ≥ 3 at biopsy). Given the relatively low number of cases with GG1 and GG2, evaluation of the relevance of all three PET parameters in the prediction of biopsy upgrading in larger populations of patients with GG ≤ 2, classified as low- or intermediate-risk at diagnosis, is required.

The inclusion of substantial numbers of patients undergoing either [^68^ Ga]Ga-PSMA-11 and [^18^F]PSMA-1007 in this study enabled in-between assessment of these radioligands regarding of the predictive value of PSMA PET parameters. No significant differences in median values of SUV_max_ and PSMA_total_ were observed per ISUP GG. These findings contradict the results previously reported by Kuten and colleagues, who reported significantly higher median SUVmax in the primary dominant intraprostatic tumors for [^18^F]PSMA-1007 compared with [^68^ Ga]Ga-PSMA-11 (8.7 vs 6.9, *p* = 0.002) [25]. Huang and colleagues also reported contradicting results in their meta-analysis, describing lesion SUV_max_ of [^18^F]PSMA-1007 was significantly higher than [^68^ Ga]Ga-PSMA-11 [26]. When adjusting for these radioligands on logistic regression, the odds of ISUP GG ≥ 4, were not statistically significant for all three parameters. However, for ISUP GG2 and GG3, significant differences in median PSMA_volume_ were observed. This may suggest estimation of this parameter is susceptible to in-between radioligand differences. An important limitation of this sub-analysis is that the majority of [^18^F]PSMA-1007 PET/CT were performed at 1 hospital (176/200, [88%]), and therefore interobserver variability as a confounder cannot be excluded. Nevertheless, at multivariable analysis, PSMA_volume_ remained significantly associated with ISUP GG ≥ 4, adjusting for confounders including radioligand and hospital, which shows its clinical relevance.

Our findings emphasize the complexity of in-between radioligand quantitative parameters; reflected by the wide IQR observed of all three parameters per ISUP GG. However, it is known that SUV values can be influenced by several factors such as time of SUV evaluation (injection-to-midacquisition time), scanner type, body size as well as techniques used in reconstruction [27]. Partly due to these limitations, the PRIMARY score (1 to 5) was developed using parameters beyond solely quantitative parameters, including a combination of pattern, zonal location and SUV_max_ (using a threshold of ≥ 12). High SUV_max_ ≥ 12 represents the top score (PRIMARY score of 5), because of its observed 100% specificity of significant malignancy [28]. However, the PRIMARY score has been developed as a risk score to assist diagnosis of clinically significant PCa. The proposed subclassification of SUV_max_ in this study can be complimentary to the PRIMARY score, as it provides additional information regarding the aggressiveness of the cancer. For instance, among patients with SUV_max_ > 28, 50% had ISUP GG ≥ 4 at surgical pathology, whereas this accounted for 11.6% of patients with SUV_max_ ≤ 6.5, respectively. In conclusion, if quantitative PET parameters are used for PCa risk prediction, the adoption of clinically relevant thresholds instead of a single numeric values are recommended, as this may lead to more accurate and reproducible predictions. The proposed clinically meaningful thresholds in this study showing to be related to ISUP GG at histopathology, providing additive information to other classification systems such as the PRIMARY score.

Besides their association with surgical ISUP GG, PSMA PET parameters have shown their potential to assist in prediction of presence of pelvic LNI. Muehlematter and colleagues showed significant higher median values in PSMA_volume_ and PSMA_total_ comparing patients with and without LNI at histopathological evaluation and this was confirmed at external validation [29]. In addition, Laudicella and colleagues showed PSMA_total_ and PSMA_volume_ to be significantly associated with pathological T stage after RP. They reported that using PSMA_total_ and PSMA_volume_ for the prediction of extraprostatic extension resulted in AUCs of 71% and 72%, respectively. By using their proposed cutoff of 24.6 g/ml x cm^3^ for PSMA_total_ and 4.41 cm^3^ for PSMA_volume_, sensitivity for the detection of EPE of 71% was reached [6]. However, this study is limited by its single-center nature and small sample size. Lastly, PSMA whole body uptake (total volume of PSMA-avid tumor) has been shown to have a direct and positive correlation with serum PSA values in prostate cancer patients with biochemical recurrence [30]. Although outside of the scope of current study, these preliminary findings regarding the predictive value of PSMA_total_ and PSMA_volume_ for local tumor stage and presence of LNI should be validated using large multi-center and multi-tracer patient populations. In this future study, focus should also be on identification of clinically relevant and reproducible thresholds for accurate predictive modelling among different patient populations. A pragmatic subclassification, as proposed in this study, could account for the variability regarding uptake parameters, and validation of our classification system in external cohorts is crucial to answer this question. In addition, future studies should also focus on the association between PSMA PET parameters and oncological outcomes including biochemical recurrence and development of metastastatic disease, which have been described previously [31, 32]. Unfortunately, this study is limited by the lack of data on follow-up and recurrence and these outcome parameters where therefore not evaluated.

Although our study has several strengths, such as a multicenter international study with one of the largest series of patients available describing the predictive value of PSMA PET quantitative parameters using different radioligands, it is not devoid of limitations. First, our study did not include central review or second reading of PSMA PET/CT. This could potentially have introduced interobserver variability. Second, we did not include intra-individual comparisons between different tracers, and although we adjusted for potential confounders on multivariate analysis, this could introduce selection bias. Third, no restrictions were used regarding type of radioligands, scanners as well as used software, which could also have led to information bias. However, the incorporation of different protocols and scanners may also be seen as a strength, as the incorporation of this heterogeneity potentially enables more robust estimations, and the variability reflects the real-world clinical situation. In addition, data regarding the location of PSMA uptake in the prostate, physiological PSMA uptake in non-malignant tissue and scoring systems integrating this information (e.g. PSMA expression V score and the PRIMARY score), were unfortunately not available in this study [28, 33]. Lastly, in-between hospital differences in selection of patients for PSMA PET/CT as well as the lack of central histopathological review. This could explain hospital to be significantly associated with ISUP GG ≥ 4 on multivariable analysis, potentially introducing selection and information bias, which could limit the generalizability of the results.

## Conclusions

We demonstrated that PSMA PET parameters SUV_max_, PSMA_volume_ and PSMA_total_ are associated with ISUP GG found at final histopathological evaluation. Our results suggest a robust classification system with clinically relevant thresholds, which has the potential to assist in prostate cancer risk stratification in daily clinical practice.

## Supplementary Information

Below is the link to the electronic supplementary material.

ESM 1 (DOCX 106 KB)

## Data Availability

The datasets generated during and/or analysed during the current study are available from the corresponding author on reasonable request.
