# Supplementary material for: The association of quantitative PSMA PET parameters with pathologic ISUP grade: an international multicenter analysis
Source: Eur J Nucl Med Mol Imaging. 2024 Aug 1;52(1):314–25. doi: 10.1007/s00259-024-06847-y (PMC11599533; doi:10.1007/s00259-024-06847-y)
Supplement: Supplementary file 1 — (DOCX 106 KB) [file 259_2024_6847_MOESM1_ESM.docx]

**Supplementals**

**Table 1. Local PSMA PET protocols**

| **Hospital 1** |  |
| --- | --- |
| **Radioligand preparation** | The [^68^Ga]Ga-PSMA-11 was prepared using a GMP (GoodManufacturing Process) grade 68Ge/68Ga generator (Eckert& Ziegler Strahlen, Berlin, Germany) and a semiautomated synthesis module (ITG, Munich, Germany). Each synthesis was performed according to manufacturer instructions using PSMA-11 ligand (ABX, Radeberg, Germany). |
| **Acquisition and Image reconstruction** | Images were acquired from the skull vertex to the midthigh using a Biograph CT40 scanner or a Gemini TF64 slice scanner (Philips, Best, The Netherlands). Intra-venous 1.5 MBq/kg 68Ga-PSMA-11 was administered followed by 500 ml saline. PET images were acquired 60minutes after injection. Low dose CT was done directly after PET. Images were acquired according to EANM (European Association of Nuclear Medicine) criteria.  Reconstruction and post-filtering: PET reconstructions were made using the scanner’s default Ordered Subset Expectation Maximization (OSEM) reconstruction algorithm with 33 subsets, 3 iterations, matrix size of 144×144, and voxels of 4×4×4 mm. No Gaussian filter was applied. The reconstruction was corrected for geometrical response and detector efficiency (normalization), random coincidences, scatter and attenuation. |
| **Nuclear physician experience** | >5 years and >500 scans |
| **Accreditation** | EARL 1 |

| **Hospital 2** |  |
| --- | --- |
| **Radioligand preparation** | [^18^F]PSMA-1007 was synthesized at the Radboud University  Medical Center translational medicine cyclotron facility.  Reagent kit and PSMA-1007 precursor were obtained from  ABX (Radeberg, Germany). |
| **Acquisition and Image reconstruction** | Images were obtained from skullbase to mid-thigh. PET/CT scans were performed on a Philips Gemini 64TF (Eindhoven, The Netherlands) or a Siemens Biograph Vision 450 (Erlangen, Germany). An fixed dose of approximately 250 MBq of [^18^F]PSMA-1007 was administered intravenously, acquisition started approximately 90 min post injection.  Patients were hyperhydrated and voided before the scan.  Reconstruction and post-filtering: Iterative+TOF with 2 iterations, Gaussian filter, FWHM 7.0mm, image size 220. Voxel size is 5 mm. |
| **Nuclear physician experience** | >5 years and >500 scans |
| **Accreditation** | EARL, in this paper EARL 1 reconstructions were used for measurements |

| **Hospital 3** |  |
| --- | --- |
| **Radioligand preparation** | [^68^Ga]Ga-PSMA-11 and [^18^F]PSMA-1007 were employed for PET imaging. Tracer precursors (PSMA-11 and PSMA-1007) were obtained from ABX advanced biochemical compounds (ABX GmbH). [^18^F]PSMA-1007 and [^68^Ga]Ga-PSMA-11 were synthesized on site using a kit-based approach on automated platforms with comprehensive pH, radiochemical, chemical, and radionuclide purity control tests. Intravenous administration was body-weight–dependent (2 MBq/kg of body weight). |
| **Acquisition and Image reconstruction** | Image acquisition (from the vertex to thigh) started 60 minutes after injection. Patients were asked to void their bladder before imaging. PET/CT was used to acquire coregistrated images. Either low-dose or full-dose CT acquisitions were obtained directly before PET acquisition using a Biograph mCT (Siemens Healthineers, Knoxville, TN, United States). PET emission data were attenuation corrected by help of the CT data and iteratively reconstructed with time-of-flight information and point-spread function correction (HD PET). PET/MRI examination was performed with an integrated 3.0-T Biograph mMR scanner (Siemens Healthineers), and simultaneous PET and 3D Dixon-volumetric interpolated breath-hold examination (VIBE) sequences for MRI-based scatter correction were performed, followed by a standardized whole-body MRI protocol.  Voxel size, reconstruction and post-filtering: For both [^18^F]PSMA-1007 and [^68^Ga]Ga-PSMA-11, attenuation corrected PET images were reconstructed iteratively using three dimensional ordinary Poisson ordered subset expectation maximization algorithm with combined time-of-flight and point-spread function modelling with 3 iterations and 21 subsets. Images were reconstructed into a 200×200 tranverse matrix and a voxel size of 3.3×3.3×3.0 mm. Standard corrections for scatter, randoms, and normalization were applied as well as an isotropic post-reconstruction 4-mm Gaussian filter. |
| **Nuclear physician experience** | >5 years and >1000 scans |
| **Accreditation** | EARL 1 and 2 |

| **Hospital 5** |  |
| --- | --- |
| **Radioligand preparation** | The [^68^Ga]Ga-PSMA-11 was prepared using a GMP (Good Manufacturing Process) grade 68Ge/68Ga generator (Eckert& Ziegler Strahlen, Berlin, Germany) and a semiautomated synthesis module (ITG, Munich, Germany). Each synthesis was performed according to manufacturer instructions using PSMA-11 ligand (ABX, Radeberg, Germany). |
| **Acquisition and Image reconstruction** | Images were acquired from the vertex to the midthigh using a scanner GE 710 (General Electric, USA). Intra-venous 185 MBq [^68^Ga]Ga-PSMA-11 was administered followed by 500 ml saline. PET images were acquired 60 minutes after injection. Low dose CT was done before PET. Images were acquired according to EANM (European Association of Nuclear Medicine) guidelines.  Voxel size: 2.7344x2.7344x3.2700 mm^3^  Reconstruction: VPFXS (OSEM+PSF+TOF) 3 interactions, 18 subsets.  Post-filtering: Filter type standard, filter cutoff/width 6.5 mm |
| **Nuclear physician experience** | >5 years and >500 scans |
| **Accreditation** | No accreditation |

| **Hospital 6** |  |
| --- | --- |
| **Radioligand preparation** | The [^18^F]PSMA-1007 injection was prepared using an automated synthesis module, Synthera® (IBA, Louvain-La-Neuve, Belgium). The single-use reaction cassette (IFP), chemical precursor and reagents are manufactured (ABX, Radeberg, Germany) according to GMP (Good Manufacturing Practice) requirements, intended for manufacture of radiopharmaceutical preparation. The product injection solution meets acceptance criteria compliant with European Pharmacopoeia (Eur. Ph.) standards. |
| **Acquisition and Image reconstruction** | Pre-examination fasting was not required. Injected activity of 18F-PSMA-1007 was calculated according to patient’s weight (6.5-11.0 mCi). After intravenous injection, patients were requested to take a rest in preparation room for a minimum uptake period of 90 minutes before scanning. Whole-body scans were acquired after urination, spanning from base of skull to upper thigh. Patients performed normal breathing with both arms positioned above head. Non-contrast CT was performed (120 kVp, 90 mAs, pitch 0.8 and rotation time 0.5 second) followed by PET acquisition (static bed, 2 minutes per bed). PET images were reconstructed using parameters optimized for small lesion depiction (4 iterations 4 subsets, gaussian filter 5 mm at FWHM, voxel size 1.65 x 1.65 x 1.5 mm, point spread function and time-of-flight options enabled. |
| **Nuclear physician experience** | >5 years and >500 scans |
| **Accreditation** | No accreditation |

| **Hospital 7** |  |
| --- | --- |
| **Radioligand preparation** | [^68^Ga]Ga-PSMA-11 will be synthesized in the radiochemistry laboratory of the Division of Nuclear Medicine of the European Institute of Oncology (IEO), Milan. Gallium-68 will be produced with 68Ge/68Ga generator (GalliaPharm 68Ge/68Ga generator, Eckert & Ziegler, Germany). 68Ga-PSMA-HBEDCC(Glu-NH-CO-NH-Lys-(Ahx)-[[68Ga]Ga(N,N′-bis-[2-hydroxy-5-(carboxyethyl)benzyl]ethylenediamine-N,N′-diacetic-acid]) (68Ga-PSMA-11) will be prepared in a similar procedure as described by Eder et al. and transferred to cassette-based automated synthesis module.[1] The whole procedure was performed in accordance with the Good Manufacturing Practices (GMP). The system provides a final report, and the resulting solution (final product) is subjected to standard quality controls. |
| **Acquisition and Image reconstruction** | In accordance with procedure guidelines all patients will receive intravenously a dose of 2 MBq/Kg ± 0.2 MBq of [^68^Ga]Ga-PSMA-11. Patients will receive intravenously hydration (0.5 L, saline solution) during uptake. No preparation before the procedure is required.  [^68^Ga]Ga-PSMA-11 PET/CT will be performed in accordance with a standard technique.[2] All patients will undergo PET/CT scan in a dedicated tomograph (GE DMI DR). An attenuation-corrected whole-body scan (vertex to mid thighs) 2 min per bed position starting 60-90 min after tracer injection will be acquired. A low-dose CT scan will be performed for attenuation correction of the PET emission data and for anatomical correlation. |
| **Nuclear physician experience** | >5 years and >500 scans |
| **Accreditation** | EARL 1 |

**Figure 1. Box plots of PSMA_total_ (a), SUV_max_ (b) and PSMA_volume_ (c) per surgical ISUP grade group**

**a.**


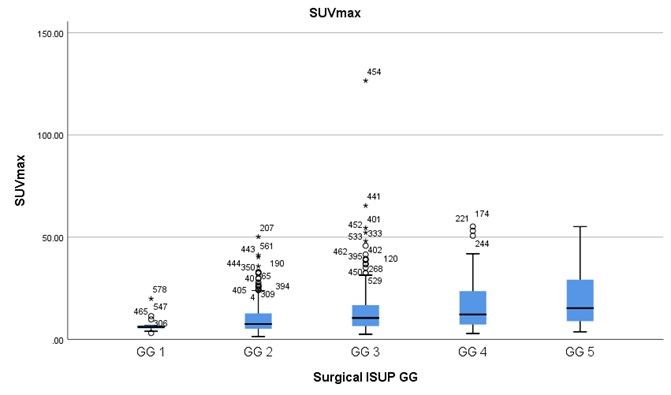


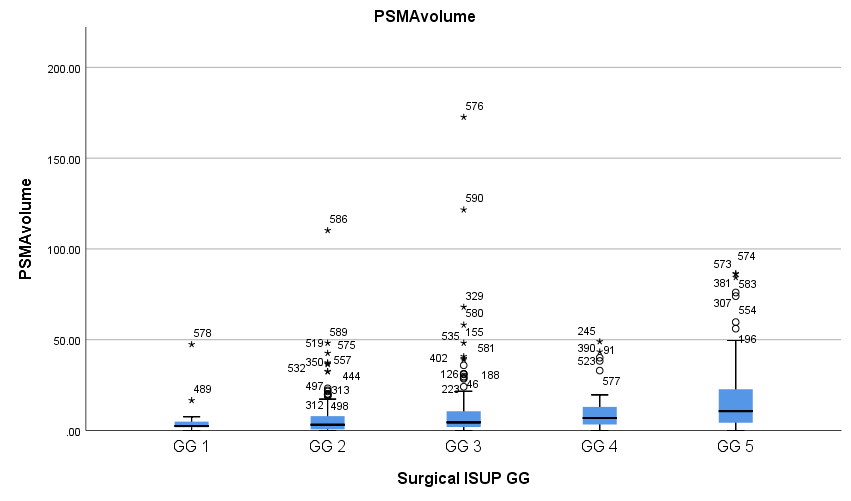
**b.**


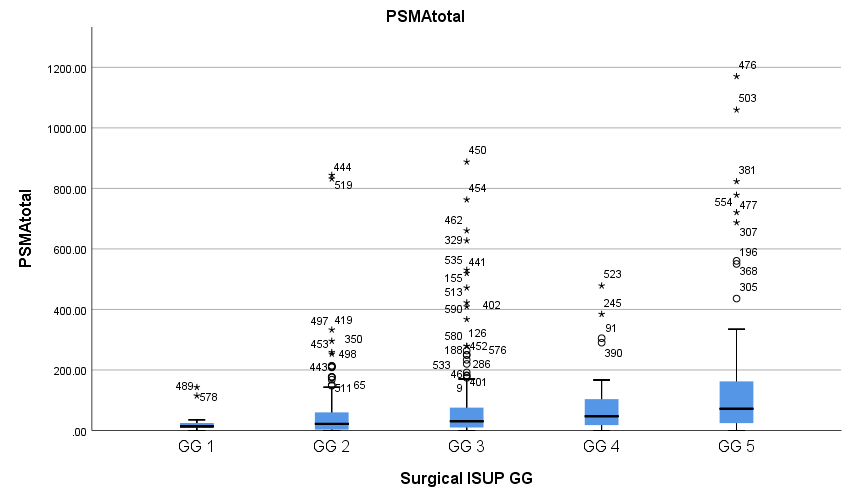
**c.**

**Table 2. Comparison of median values in PSMA PET parameters per ISUP grade group for [^68^Ga]Ga-PSMA-11 and [^18^F]PSMA-1007**

| **Grade group** | **Parameter** | **[^68^Ga]Ga-PSMA-11**  **Median, IQR** | **[^18^F]PSMA-1007**  **Median, IQR** | ***P*** |
| --- | --- | --- | --- | --- |
| **GG1 (N=14)** | **SUV_max_** | 6.6 (5.5 – 11.0) | 5.7 (4.4 – 6.4) | 0.18 |
|  | **PSMA_volume_** | 2.8 (1.9 – 13.7) | 2.0 (0.7 – 6.1) | 0.44 |
|  | **PSMA_total_** | 18.5 (11.0 – 92.3) | 8.7 (3.0 – 27.8) | 0.28 |
| **GG2 (N=216)** | **SUV_max_** | 7.0 (4.8 – 13.2) | 8.4 (5.9 – 11.7) | 0.42 |
|  | **PSMA_volume_** | 2.3 (0.4 – 7.7) | 5.6 (1.4 – 9.4) | **0.03** |
|  | **PSMA_total_** | 15.3 (1.7 – 66.7) | 29.3 (7.0 – 54.1) | 0.06 |
| **GG3 (N=211)** | **SUV_max_** | 10.4 (5.7 – 15.1) | 11.3 (7.2 – 17.7) | 0.21 |
|  | **PSMA_volume_** | 3.5 (1.2 – 8.9) | 6.7 (3.7 – 13.6) | **0.03** |
|  | **PSMA_total_** | 26.0 (6.1 – 67.7) | 36.9 (18.8 – 79.1) | 0.06 |
| **GG4 (N=54)** | **SUV_max_** | 10.9 (6.4 – 22.7) | 14.6 (7.6 – 25.3) | 0.32 |
|  | **PSMA_volume_** | 7.3 (1.3 – 16.4) | 7.7 (3.6 – 12.7) | 0.53 |
|  | **PSMA_total_** | 49.2 (5.8 – 108.0) | 63.7 (21.8 – 103.6) | 0.67 |
| **GG5 (N=70)** | **SUV_max_** | 14.9 (9.1 – 29.9) | 13.6 (7.7 – 27.5) | 0.49 |
|  | **PSMA_volume_** | 10.7 (4.1 – 29.3) | 9.0 (4.0 – 16.7) | 0.43 |
|  | **PSMA_total_** | 80.9 (26.9 – 227.8) | 56.9 (20.9 – 119.3) | 0.19 |

**P values represent Mann Whitney U test**

**Table 3. Uni- and multivariable logistic regression analysis of high-risk surgical ISUP grade group (4 and 5) for PSMA_total_ (a), SUV_max_ (b) and PSMA_volume_ (c)**

| **Variable** | **OR (95%CI)** |
| --- | --- |
| SUV_max_ | 1.045 (1.027 – 1.064) |
| PSMA_volume_ | 1.026 (1.013 – 1.039) |
| PSMA_total_ | 1.003 (1.002 – 1.004) |

**Table 4. Multivariable logistic regression analysis of high-risk surgical ISUP grade group assessing the impact of hospital and radioligand type on model discrimination**

|  | **Model A**  **OR (95%CI)** | **Model B**  **OR (95%CI)** | **Model C**  **OR (95%CI)** | **Model D**  **OR (95%CI)** |
| --- | --- | --- | --- | --- |
| SUV_max_ |  | 1.031 (1.009 – 1.054) | 1.029 (1.007 – 1.053) | 1.020 (0.997 – 1.044) |
| PSMA_volume_ |  | 1.011 (0.996 – 1.028) | 1.012 (0.996 – 1.027) | 0.997 (0.979 – 1.014) |
| PSMA_total_ |  | 1.001 (0.999 – 1.003) | 1.0014 (0.9995 – 1.0034) | 1.005 (1.002 – 1.008) |
| Radioligand  [^68^Ga]Ga-PSMA-11  [^18^F]PSMA-1007 |  |  | Ref  1.53 (0.98 – 2.38) |  |
| PSA | 0.99 (0.97 – 1.001) | 0.99 (0.97 – 1.001) | 0.99 ( 0.970 – 1.002) | 0.97 (0.95 – 0.99) |
| Clinical stage  T1  T2  T3 | Ref  1.92 (1.22 – 3.04)  3.66 (2.00 – 6.66) | Ref  1.97 (1.22 – 3.22)  3.94 (2.10 – 7.39) | Ref  1.96 (1.21 – 3.21)  3.98 (2.11 – 7.47) | Ref  1.66 (1.01– 2.74)  2.83 (1.48 – 5.39) |
| Hospital  1  2  3  4  5-6-7 |  |  |  | Ref  1.54 ( 0.87 – 2.79)  2.11 (0.99 – 4.49)  0.09 (0.02 – 0.34)  1.63 ( 0.75 – 3.48) |
| **AUC (%) (95%CI)** | **65 (59 – 71)** | **71 (66 – 76)** | **71 (66 – 77)** | **75 (70 – 80)** |

**Table 5. Univariable logistic regression analysis assessing upgrading in patients with biopsy GG≤4 at final histopathology for all three PSMA parameters (continuous and categorical)**

|  | **OR (95%CI)** |
| --- | --- |
| SUV_max_ | 1.024 (1.004 – 1.043) |
| SUV_max_  0-6.5  6.5-15  15-28  >28 | ref  1.31 (0.75 – 2.31)  1.82 (0.96 – 3.46)  2.64 (1.23 – 5.67) |
| PSMA_volume_ | 1.023 (1.008 – 1.038) |
| PSMA_volume_  0-2  2-9  9-20  >20 | ref  2.85 (1.53 – 5.33)  2.52 (1.21 – 5.23)  4.57 (2.00 – 10.46) |
| PSMA_total_ | 1.002 (1.000 – 1.004) |
| PSMA_total_  0-12  12-98  >98 | ref  2.34 (1.31 – 4.18)  2.42 (1.19 – 4.92) |

**Table 6. Multivariable logistic regression analysis upgrading in patients with biopsy ISUP GG1-4 at final histopathology**

|  | **OR (95%CI)** | **AUC (95%CI)** |
| --- | --- | --- |
| **SUV_max_**  **PSMA_volume_**  **PSMA_total_** | 1.023 (0.999 – 1.048)  1.027 (1.007 – 1.049)  0.998 (0.994 – 1.001) | 61 (55 –67) |

**Table 7. Univariable logistic regression analysis downgrading at surgical pathology in patients with biopsy ISUP GG≥2 for all three PSMA parameters (continuous and categorical)**

|  | **OR (95%CI)** |
| --- | --- |
| SUV_max_ | 0.98 (0.983 – 1.012) |
| SUV_max_  0-6.5  6.5-15  15-28  >28 | ref  1.33 (0.88 - 2.02)  1.42 (0.86 – 2.32)  0.57 (0.28 – 1.13) |
| PSMA_volume_ | 0.983 (0.968 – 0.997) |
| PSMA_volume_  0-2  2-9  9-20  >20 | ref   - 1. (0.67 – 1.53)   0.79 (0.48 – 1.30)  0.65 (0.34 – 1.24) |
| PSMA_total_ | 1.00 (0.998 – 1.001) |
| PSMA_total_  0-12  12-98  >98 | ref  1.00 (0.67 – 1.49)  0.89 (0.54 – 1.48) |

**Table 8. Multivariable logistic regression analysis downgrading at surgical pathology in patients with biopsy ISUP GG≥2**

|  | **OR (95%CI)** | **AUC (95%CI)** |
| --- | --- | --- |
| **SUV_max_**  **PSMA_volume_**  **PSMA_total_** | 1.001 (0.983 – 1.020)  0.973 (0.952 – 0.994)  1.001 (0.999 – 1.003) | 0.55 (0.50 – 0.60) |

**References**

1. Eder M, Schafer M, Bauder-Wust U, Hull WE, Wangler C, Mier W, et al. 68Ga-complex lipophilicity and the targeting property of a urea-based PSMA inhibitor for PET imaging. Bioconjug Chem. 2012;23:688-97. doi:10.1021/bc200279b.

2. Fendler WP, Eiber M, Beheshti M, Bomanji J, Ceci F, Cho S, et al. (68)Ga-PSMA PET/CT: Joint EANM and SNMMI procedure guideline for prostate cancer imaging: version 1.0. Eur J Nucl Med Mol Imaging. 2017;44:1014-24. doi:10.1007/s00259-017-3670-z.
